# Case Report: Primary adrenal insufficiency due to bilateral adrenal infarction and antiphospholipid syndrome in Covid19 - A complicate case of cardiogenic shock

**DOI:** 10.3389/fendo.2025.1554009

**Published:** 2025-04-02

**Authors:** Giuseppe Fischetti, Antonella Barbone, Lorenzo Giovannico, Giuseppe Palma, Federica Mazzone, Nicola Di Bari, Domenico Parigino, Luca Savino, Ludovico Di Gioia, Irene Caruso, Aline Maria Silva, Aldo Domenico Milano, Massimo Padalino, Tomaso Bottio, Francesco Giorgino, Sebastio Perrini

**Affiliations:** ^1^ Department of Precision and Regenerative Medicine and Ionian Area (DiMePRe-J), Cardiac Surgery Unit, University of Bari “Aldo Moro”, Bari, Italy; ^2^ Department of Precision and Regenerative Medicine and Ionian Area, Section of Internal Medicine, Endocrinology, Andrology and Metabolic Disease, University of Bari Aldo Moro, Bari, Italy; ^3^ Section of Endocrinology, Department of Medicine and Surgery, Libera Università Mediterranea (LUM) University, Casamassima, Bari, Italy

**Keywords:** case report, primary adrenal insufficiency (PAI), catastrophic antiphospholipid syndrome (CAPS), bilateral adrenal hemorrhage, hormone replacement therapy

## Abstract

We report a clinical case of multifactorial shock and primary adrenal insufficiency (PAI), caused by bilateral adrenal hemorrhage in the context of catastrophic antiphospholipid syndrome (CAPS) triggered by a COVID-19 infection. A 54-year-old woman was admitted with cardiogenic shock, presenting with severe cardiac dysfunction, neurological alterations, and systemic embolism. Despite initial treatment for suspected septic shock, her condition deteriorated, with bilateral adrenal hemorrhages, markedly elevated adrenocorticotropic hormone (ACTH) levels, low cortisol, and positive antiphospholipid antibodies, leading to the diagnosis of PAI. A multidisciplinary approach, including endocrinology and cardiology expertise, enabled the prompt initiation of hydrocortisone and anticoagulant therapy, which significantly improved her hemodynamic stability and overall clinical status. At follow-up, partial recovery of left ventricular function was observed, although residual cardiac dysfunction persisted. This case highlights the diagnostic challenges associated with CAPS, a rare autoimmune disorder with life-threatening manifestations, including PAI due to adrenal infarction. The overlapping symptoms of CAPS and septic shock often delay diagnosis, underscoring the importance of early recognition of adrenal involvement in patients with CAPS. Furthermore, the patient clinical history, including anticoagulant withdrawal and previous thrombotic events, suggests a need for heightened vigilance in similar cases. In recent years, strong evidence has emerged on the similarities between CAPS and COVID-19, particularly related to the immungenic power of this viral infection and hypercoagulability, but it is also considered that COVID-19 can trigger CAPS. Our findings emphasize the critical role of a coordinated multidisciplinary approach in managing complex CAPS presentations and underline the importance of timely hormone replacement and anticoagulation to improve outcomes in PAI associated with adrenal hemorrhage.

## Introduction

Antiphospholipid syndrome (APS) is an autoimmune disorder that presents with sudden onset of widespread thrombosis due to uncontrolled complement activation in individuals with antiphospholipid antibodies (aPLs). The aberrant activation is triggered by factors such as infection, malignancy or surgery, and results in widespread ischemic damage and the onset of multiple organ failure (1).

Since the outbreak of the SARS-CoV-2 virus pandemic, an important relationship between COVID-19 and antiphospholipid antibody syndrome has been observed. This relationship is strongly supported by the hypercoagulation state observed in patients with acute COVID-19 and the critical role of anticoagulant treatment in affected individuals. In addition, patients with COVID-19 exhibited antiphospholipid antibodies of different types after infection, underscoring the hypothesis that SARS-CoV-2 infection may be an important trigger for APS, also in light of the now known data on the autoimmune effect brought about by this type of infection (2).

Catastrophic antiphospholipid syndrome (CAPS) is a life-threatening condition with a high mortality rate even in case of prompt diagnosis and intervention (3). CAPS has also been shown to have overlapping features with severe COVID-19, such as cytokine storm and multiorgan failure (2).

One of the complications of catastrophic antiphospholipid antibody syndrome is primary adrenal insufficiency (PAI) (4).

PAI is a condition characterized by inadequate production of steroid hormones (cortisol, aldosterone and androgens) by the adrenal cortex. This presents with a range of typical signs, including hypotension, electrolyte imbalances, and melanoderma, which is directly associated with the chronic, excessive stimulation of ACTH due to the absence of cortisol-mediated negative feedback on the hypothalamic-pituitary axis. The most commonly reported symptoms are fatigue, muscle weakness, loss of appetite, weight loss, and abdominal pain. In addition to a clinical evaluation, the diagnosis of adrenal insufficiency is also supported by laboratory tests, which demonstrate the presence of low cortisol and/or aldosterone levels, elevated ACTH and renin levels, hyponatremia, hyperkalemia and hypoglycemia (5).

Although it is a rare condition (approximately 0.4% of PAI cases are associated with APS), primary adrenal insufficiency represents the most common endocrine manifestation of anti-phospholipids antibody syndrome (3). It is estimated that between 10 and 26% of CAPS cases may present with adrenal insufficiency with approximately one-third of patients exhibiting adrenal involvement over the course of catastrophic APS (3).

This complication can be caused by either adrenal venous thrombosis and subsequent hemorrhagic infarction, or directly by spontaneous adrenal hemorrhage, which is often triggered by anticoagulant therapy withdrawal (6), surgery or infection (7).

We present a clinical case of multifactorial shock and PAI, driven by CAPS, in COVID-19, emphasizing the importance of its early identification and treatment of complications.

## Case

In October 2023, a 54-year-old female patient was admitted to our facility with a diagnosis of cardiogenic shock. In her medical history, the patient had undergone two pregnancies, two miscarriages and a pelvic fracture two months earlier, which had been treated with anticoagulant therapy with low-molecular-weight heparin for thromboembolic prophylaxis, for one month, and subsequently discontinued. Upon admission to the Emergency Department, the patient exhibited signs of disorientation, dyspnea, fever, and abdominal pain. A chest X-ray revealed the presence of pulmonary parenchymal opacity, thereby prompting the initiation of antibiotic therapy in light of a suspected diagnosis of pneumonia. In addition, swabbing for SARS-CoV-2 was performed and found to be positive, so appropriate isolation measures were taken. Subsequently, she was transferred to the Coronary Intensive Care Unit due to the emergence of cardiac failure-related symptoms. The echocardiogram showed severe left ventricular systolic dysfunction. The coronary angiography examination did not reveal any hemodynamically significant stenosis. Given the deterioration in her hemodynamic status, a transfer to our center was deemed necessary to explore the potential for extracorporeal membrane oxygenation (ECMO) assistance. Upon admission, the patient exhibited asthenia and displayed alternating periods of lucidity and confusion. Blood pressure was 95/50 mmHg with inotropic support provided by dobutamine 7 μg/kg/min and noradrenaline 0.5 μg/kg/min. The patient displayed a fever with a temperature of 37.9°C and an oxygen saturation of 95% which was maintained with supplemental oxygen administered through a Venturi mask at a FiO2 of 35%. Renal impairment was observed with a creatinine of 2.01 mg/dL (reference values, r.v. 0.51 - 0.95) with preserved urine output. Laboratory analysis also revealed anemia, thrombocytopenia, elevated C-reactive protein (168 mg/L, r.v. <4), procalcitonin (218.8 ng/mL, r.v. <0.5), and D-dimer levels (3429 μg/L, r.v. <500), NT-proBNP (19,745 pg/mL, r.v. <166), in addition to increasing troponin levels, peaking at 753 pg/mL (r.v. <51) with fluctuating pattern. Blood culture was positive for S. epidermidis, led to the initiation of targeted antibiotic therapy.

The electrocardiogram revealed sinus tachycardia at 110 bpm. Transthoracic echocardiography showed features consistent with Takotsubo-like cardiomyopathy, characterized by a dilated left ventricle with apical and mid-segmental akinesia. This diagnosis was then confirmed by ultrasound biopsy, with histological evidence of dilatative cardiomyopathy. Ejection fraction (EF) was 25% (r.v. >55%) and global longitudinal strain (GLS) was -8%. Levosimendan administration transiently improved contractility, yet the patient remained reliant on inotropic support, reaching Interagency Registry for Mechanically Assisted Circulatory Support (INTERMACS) score 3. This tool evaluates the severity and prognosis of patients with advanced heart failure who are receiving mechanical circulatory support (8). In this clinical case, a score of 3 indicates a severe picture of hemodynamic impairment.

**Figure 1:**
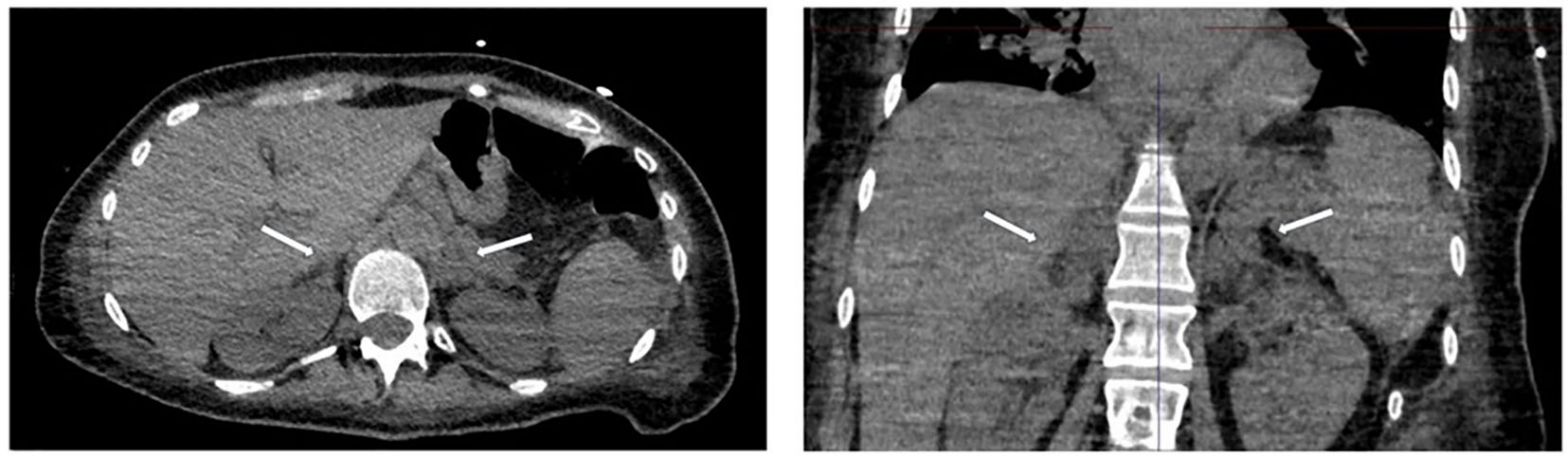
Transverse (left) and coronal (right) CT images of the abdomen, showing bilateral adrenal hemorrhage.

Meanwhile, within one week, neurological symptoms progressively worsened. A comprehensive total body computed tomography (CT) scan was performed due to abdominal pain, the patient’s shock state, and neurological impairment. The imaging revealed pulmonary, splenic, and renal emboli, along with bilateral adrenal hemorrhages. The CT images (**Figure 1**) showed bilateral adrenal gland enlargement with loss of their normal Y- or V-shaped morphology. The glands appeared bulky and diffusely thickened, exhibiting increased attenuation on non-contrast imaging. The adrenal parenchyma was heterogeneously hyperdense, with a density of ~60 Hounsfield Units (HU), consistent with acute hemorrhagic content. The contours were slightly irregular but remained distinguishable from the surrounding tissues. No evidence of calcifications or chronic remodeling was observed. Periadrenal fat stranding was present, suggesting associated inflammatory or edematous changes. These findings were characteristic of bilateral adrenal hemorrhage, requiring clinical correlation for adrenal insufficiency assessment.

In fact, blood tests revealed hyponatremia (132 mg/dL, r.v. 135 - 145 mg/dL), low cortisol (4 μg/L, r.v. 9 - 23 μg/L), and elevated adrenocorticotropic hormone (ACTH) levels (437 pg/mL, r.v. 5 - 55 pg/mL), which confirmed the diagnosis of primary adrenal insufficiency. However, serum potassium levels were within normal limits (3.9 mg/dL, r.v. 3.5 - 5.5 mg/dL). Anticoagulant therapy with enoxaparin was started. An endocrine referral was requested, and hormone replacement therapy with hydrocortisone was promptly initiated. A 100 mg hydrocortisone bolus was administered intravenously, followed by 50 mg every 6 hours. Endocrine evaluation revealed skin hyperpigmentation consistent with ACTH-induced melanogenesis, suggesting a possible chronic PAI that was subsequently exacerbated. Following the initiation of corticosteroid replacement therapy, the patient’s neurological status and blood pressure improved significantly, along with left EF 45% at discharge, allowing the discontinuation of the supportive amine therapy. This was further supported by the continuation of targeted antibiotic therapy and fluid support for shock management.

Further serological investigations showed positive antinuclear antibody titers (1:160), lupus anticoagulant, anticardiolipin antibodies (IgG >640 GPLU/mL), and antibeta-2-glycoprotein-I antibodies (IgG >867 CU/m).

Subsequently, the patient was transferred to the endocrinology department, where she underwent the 21-hydroxylase antibody assay (negative results), confirming the diagnostic hypothesis of CAPS-associated PAI. During approximately two weeks of hospitalization, hydrocortisone therapy was gradually tapered by transitioning to oral tablets at a daily dose of 25 mg. Upon discharge, fludrocortisone therapy was initiated at a dosage of 0.1 mg daily.

At the 1-month follow-up, the patient was neurologically normal and maintained eupnea while breathing room air. An echocardiogram showed normalized chamber size with mild left ventricular systolic dysfunction (EF: 48%; GLS: -16%), persistent apical and mid-segmental hypokinesis. At control tests, plasma ACTH was 15.5 pg/mL, NT-proBNP: 4,457 pg/mL, CRP: 20.1 mg/L. Plasma electrolytes normalized (sodium was 135 mg/dL, potassium was 3.6 mg/dL, chloride was 101, r.v. 98 - 107) and, on examinations prior to discharge, liver function was also normal and renal function improved (creatinine was 0.35 mg/dL). The patient also reported an improvement in her general status, especially asthenia. Moreover, upon further anamnestic re-assessment, the patient stated she had several hematochemical tests showing thrombocytopenia, not further investigated, which, together with miscarriages, would have been a useful indicator for the early diagnosis of APS.

## Discussion

Herein we present the clinical case of a patient experiencing multiple simultaneous forms of shock: septic shock secondary to a Staphylococcus epidermidis infection, cardiogenic shock due to dilated cardiomyopathy, and hypovolemic shock resulting from acute adrenal insufficiency. Initially, the condition was attributed solely to septic shock until the emergence of diffuse thrombosis and the detection of antiphospholipid antibodies (aPLs) ultimately led to the diagnosis of catastrophic antiphospholipid syndrome (CAPS). The diagnosis of CAPS is based on the following criteria: involvement of three or more organs, rapid onset within one week, histopathologic evidence of small vessel occlusion, and the presence of aPLs (6). Despite the involvement of three organs and the presence of aPLs, the diagnosis was considered probable in the absence of histopathologic confirmation. Thrombosis of the adrenal glands with bilateral hemorrhage is a recognized manifestation of APS, leading to massive hemorrhage and primary adrenal insufficiency, which may go unrecognized in the context of multiorgan failure. The role of SARS-CoV-2 infection is noteworthy; although some cases of CAPS-related PAI have been reported, few have involved active COVID-19.

Battharai et al. (9) described adrenal insufficiency linked to COVID-19 due to autoimmune adrenalitis (anti-21 hydroxylase positive, unlike our case). Marques et al. (10) also reported a similar case, but neither study assessed antiphospholipid antibodies. Another report described bilateral adrenal hemorrhage in COVID-19 without coagulopathy.

The presence of SARS-CoV-2 infection could also explain Takotsubo syndrome. Catecholamines play a crucial role in stress-induced cardiomyopathy. During a catecholamine surge, epinephrine stimulates β2-adrenergic receptors in cardiac tissue, resulting in the characteristic ultrasound finding of apical ballooning. In patients with COVID-19, elevated catecholamine levels may occur either as a compensatory mechanism to maintain circulatory stability or due to exogenous intravenous infusions of adrenaline and norepinephrine (as in our patient) to ensure adequate perfusion of vital organs. In these circumstances, the increased catecholamine levels can contribute to the development of stress-induced cardiomyopathy, leading to transient myocardial dysfunction manifested as apical ballooning. Additionally, impaired coronary perfusion or vasospasm may further exacerbate this transient cardiac dysfunction, similar to that observed in other critical conditions (11). Antiphospholipid antibodies in COVID-19 were first observed in early pandemic reports linking APS-like thrombosis with infection (12). Our case, marked by antiphospholipid autoantibody positivity, supports a diagnosis of CAPS-related primary adrenal insufficiency, potentially triggered by SARS-CoV-2 infection or the discontinuation of anticoagulant therapy following a hip fracture.

A late diagnosis of primary adrenal insufficiency due to bilateral adrenal hemorrhage was made, and this delay contributed to the aggravation of the patient’s hemodinamic status. This case highlights the complexity of CAPS diagnosis, which can present with a wide range of symptoms and mimic other conditions. The diagnosis of CAPS requires the following manifestations: involvement of three or more organs, rapid onset (within one week), histopathological evidence of small vessel occlusion, and the presence of aPLs (6). Despite the involvement of three organs and the presence of aPLs, the diagnosis was considered probable as histopathological confirmation was lacking.

Another typical feature of antiphospholipid antibody syndrome is thrombocytopenia: this condition is common in patients with the anti-phospholipid antibody syndrome and is usually mild (13). In our clinical case, upon further anamnestic re-assessment, the patient stated she had several hematochemical tests showing thrombocytopenia, not further investigated, which, together with miscarriages, would have been a useful indicator for the early diagnosis of APS.

According to an online registry of 500 cases, cardiac involvement in CAPS is observed in half of all cases, mainly due to myocardial infarction or valvulopathy (14, 15). Myocardial infarction is the most common complication, accounting for 25% of cases. The underlying mechanisms involve either arterial or venous thrombi within the coronary arteries or microvascular thrombosis affecting the coronary microcirculation. For instance, Cranley et al. described a 51-year-old woman with a history of myocardial infarction and unobstructed coronary arteries on angiography who developed bilateral adrenal hemorrhage resulting in acute adrenal insufficiency and microvascular thrombotic renal disease, leading to the diagnosis of CAPS (15). In addition, valvular involvement, commonly associated with systemic lupus erythematosus (SLE), may manifest as Libman-Sacks endocarditis, leading to valvular regurgitation or stenosis. Cardiomyopathy is less commonly described, and may develop as a result of coronary artery occlusion or significant valvulopathy. However, rare case reports have identified CAPS patients with dilated cardiomyopathy, a normal coronary angiogram (as in our patient), negative viral serologic tests, and no other identifiable cause of heart failure on routine testing (14). Congestive heart failure is a common cardiac complication in CAPS, which can result from myocardial damage caused by thrombotic events or immune-mediated inflammation (15).

Thrombosis of the adrenal glands causing bilateral hemorrhage is a well-known presentation of APS. It is characterized by massive hemorrhage and primary adrenal insufficiency; and may go unrecognized in the context of multiorgan failure (16, 17). Heart failure is a challenging clinical condition resulting from structural or functional abnormalities, caused by disorders within the pericardium, myocardium, or endocardium, or from metabolic irregularities. Treatment protocols often follow a standardized approach, but elucidating the underlying mechanism of heart failure remains paramount for effective short- and long-term strategies (15, 18).

Management of cardiac complications in CAPS requires a comprehensive approach, including close monitoring, aggressive anticoagulation, immunosuppressive therapy, and supportive care measures. Early recognition of this rare syndrome and the treatment of complications is essential for initiating appropriate management strategies and improving patient outcomes.

The complexity of this clinical case is due to the coexistence of several pathological conditions. Cardiogenic shock, resulting from dilated cardiomyopathy, was aggravated by hypotension associated with sepsis and primary adrenal insufficiency.

The treatment of acute adrenal insufficiency resulted in a marked improvement in the patient’s clinical condition. According to the Endocrine Society guidelines, the management of adrenal crisis involves the immediate administration of 100 mg of hydrocortisone via parenteral injection, followed by appropriate fluid resuscitation. Subsequently, a continuous infusion or repeated injections totaling 200 mg of hydrocortisone per day is recommended for the first 24 hours. This regimen is designed to rapidly restore cortisol levels, which are critical for stabilizing the patient. In our case, high-dose corticosteroid treatment was maintained for several days and gradually tapered in parallel with the improvement of the infection, in accordance with the well-established principle that acute infections increase the demand for corticosteroids. Additionally, at the initial diagnosis of adrenal insufficiency, fludrocortisone therapy was not initiated because high-dose hydrocortisone provides sufficient mineralocorticoid activity. Several studies indicate that 40 mg of hydrocortisone is roughly equivalent to 100 μg of fludrocortisone, suggesting that higher doses of hydrocortisone can adequately substitute for mineralocorticoid supplementation. Therefore, during the acute phase, high-dose hydrocortisone alone is considered sufficient to address both glucocorticoid and mineralocorticoid requirements (5). In our patient’s case, the infection may have triggered the CAPS, as well as the discontinuation of anticoagulant therapy, which had been started about two months earlier due to the pelvic fracture.

This underlines the importance not only of timely diagnosis, but also of appropriate multidisciplinary care, which in our case included the cardiac surgeon, the endocrinologist, the radiologist, the infectivologist and the internist. In the specific case of our patient, hypotension caused by septic and cardiogenic shock masked the hypovolemic shock associated with acute adrenal crisis, delaying the diagnosis. This underscores the importance of considering multiple potential underlying causes in clinical presentations. A thorough differential diagnosis of shock is crucial, as various types, such as hypovolemic, cardiogenic, septic, and others, can present with similar clinical features but require different management strategies. Often, more than one condition may coexist, complicating the diagnosis and highlighting the need for a comprehensive assessment.

To facilitate the recognition of acute adrenal insufficiency in patients with shock, clinicians should consider features such as refractory hypotension despite adequate fluid resuscitation, electrolyte imbalances (particularly hyponatremia and hyperkalemia), and clinical signs including fatigue, nausea, and vomiting. Diagnostic confirmation can be obtained through cortisol measurements and ACTH stimulation tests, especially in patients with a known history of adrenal disorders or those suspected of having critical illness-related adrenal insufficiency (5).

## Data Availability

The original contributions presented in the study are included in the article/supplementary material. Further inquiries can be directed to the corresponding author.
